# Genetic Sequencing of a Bacterial Pneumonia Vaccine Produced in 1916

**DOI:** 10.3390/vaccines13050491

**Published:** 2025-05-02

**Authors:** Yongli Xiao, Sebastian M. Gygli, Tomoko Y. Steen, Jeffery K. Taubenberger

**Affiliations:** 1 Viral Pathogenesis and Evolution Section, Laboratory of Infectious Diseases, National Institute of Allergy and Infectious Diseases, National Institutes of Health, Bethesda, MD 20892, USA; sebastian.gygli@nih.gov (S.M.G.); taubenbergerj@niaid.nih.gov (J.K.T.); 2 Graduate Program in Biohazardous Threat Agents and Emerging Infectious Diseases, School of Medicine, Georgetown University, Washington, DC 20007, USA; tomoko.steen@georgetown.edu

**Keywords:** bacterial vaccine, next-generation sequencing, *Haemophilus influenzae*, *Streptococcus pyogenes*, *Enterococcus faecium*

## Abstract

**Background/Objectives**: Bacterial vaccines were first developed and used in the late 1800s to prevent chicken cholera and anthrax. Bacterial pneumonia vaccines were widely used during the 1918 influenza pandemic, despite the influenza A/H1N1 virus not yet being identified. Studies showed that bacterial pathogens, including *Haemophilus influenzae*, *Streptococcus pneumoniae*, and *Streptococcus pyogenes*, contributed significantly to fatal secondary bacterial pneumonias during the pandemic. In this study, we aimed to characterize the microbial composition of two ampules of a mixed bacterial influenza vaccine produced in 1916, which were labeled as containing killed *Bacillus influenzae*, Pneumococci, and *Streptococcus pyogenes*. **Methods**: DNA was extracted from two 1916-era vaccine ampules, and due to low DNA yields, whole genome amplification (WGA) was performed prior to construction of Illumina sequencing libraries. Deep sequencing was conducted, followed by bioinformatic analysis to identify bacterial DNA content. Consensus genomes were assembled for predominant species, and further analyzed for serotype, phylogeny, and antibiotic resistance genes. **Results**: The amount of recoverable DNA from these century-old vaccine ampules was limited. The sequencing results revealed minimal detectable *S. pneumoniae* DNA. The first ampule contained predominantly *H. influenzae* DNA, while the second vial primarily contained *Enterococcus faecium* DNA, in addition to *S. pyogenes* DNA. Consensus genomes for *H. influenzae*, *S. pyogenes*, and *E. faecium* were assembled and analyzed for serotype, phylogeny, and antibiotic resistance genes. **Conclusions**: This study presents the first genomic analysis of century-old bacterial pneumonia vaccine ampules from the 1918 influenza pandemic era. The findings provide a unique historical perspective on early vaccine formulations and highlight the limitations of early vaccine production.

## 1. Introduction

The first bacterial vaccine was developed by Louis Pasteur in 1877 to prevent cholera in chickens [1]. He later produced vaccines using weakened anthrax bacilli, which provided protection for sheep and other animals, in 1880 [2]. Emil von Behring and Shibasaburo Kitasato developed “antitoxins”, then known as serum therapy, in 1890, against both diphtheria and tetanus, which paved the way for the development of vaccines [3]. In 1911, Sir Almroth Wright led the first trial of a whole-cell pneumococcal vaccine among several thousand gold miners in South Africa [4,5]. During the 1918 influenza A/H1N1 pandemic, one of the deadliest pandemics in modern history, different bacterial vaccines were widely used, even though the viral cause of influenza had not yet been discovered. At that time, many clinicians and microbiologists believed clinical influenza was caused by a bacterium then called Pfeiffer’s bacillus or *Bacillus influenzae* (now *Haemophilus influenzae*), as it was commonly isolated from influenza cases by Richard Pfeiffer prior to the 1918 pandemic [6]. However, by 1918, growing evidence challenged the idea that Pfeiffer’s bacillus was the cause of influenza [6], including studies by J.J. Keegan [7], Edwin O. Jordan [8], and Nuzum [9]. In particular, several studies pointed to secondary bacterial infections—especially from streptococci and pneumococci—as major contributors to mortality [10,11]. 

Because of these observations, many different bacterial vaccines were developed early in the 1918 influenza pandemic to treat secondary bacterial pneumonias. Some vaccines, like those created by William H. Park in New York [12] and others at Tufts [13] and Pittsburgh [14] universities, targeted *Pfeiffer’s bacillus*. However, mixed bacterial vaccines became more common [6,15]. For example, a vaccine developed by naval medical officers in San Francisco contained Pfeiffer’s bacillus, types I, II, and III pneumococci, and streptococci [16,17]. The most widely used vaccine, by Edward C. Rosenow at the Mayo Clinic, contained a mix of pneumococci, hemolytic streptococci, *Staphylococcus aureus*, and *H. influenzae*, and was periodically adjusted to match circulating pathogens [18].

These vaccines did not target the influenza A/H1N1 virus itself, as the viral nature of influenza was unknown at the time. The effectiveness of these vaccines remains debated [15,17,19]. Some contemporary studies report little benefit [20], while others suggest Rosenow’s vaccine may have offered protection against secondary infections [15]. While these vaccines may have provided some protection against secondary bacterial infections, they could not prevent primary influenza virus infection. Effective antiviral vaccines were not developed until the 1940s [21].

Overall, bacterial vaccines have played a critical role in reducing the burden of infectious diseases caused by bacteria, leading to significant decreases in morbidity and mortality worldwide. In the current study, we analyzed two unopened glass ampules of a mixed bacterial vaccine produced by The Upjohn Company in 1916, and labeled as of containing killed *B. influenzae*, Pneumococci, and *Streptococcus pyogenes*. Applying modern next-generation sequencing (NGS) and other molecular biology techniques, we extracted and characterized the DNA content from these century-old vaccines. Our aim was to determine the DNA composition, evaluate bacterial genomic integrity, and analyze additional genomic features present in the sample. This work provides a unique glimpse into early 20th-century vaccine formulations and provides valuable insights into the microbial landscape of historical bacterial vaccines.

## 2. Materials and Methods

### 2.1. DNA Isolation

DNA isolation was performed on 550 μL of well-shaken vaccine solution twice because the total vaccine volume was ~1100 μL. The supernatant and precipitate of the vaccine solution were obtained by centrifugation at 16,000× *g* at 4 °C for 10 min. DNA isolation from the supernatant portion was performed using the following methods: (1) Phenol/chloroform extraction: One volume (550 μL) of phenol/chloroform/isoamyl alcohol (25:24:1) (MilliporeSigma, Burlington, MA, USA) was added to the obtained supernatant and vortexed for approximately 20 s, followed by centrifugation at room temperature for 5 min at 16,000× *g*. The upper aqueous phase was carefully removed and transferred to a fresh tube to proceed with ethanol precipitation. (2) A NucleoSpin cfDNA isolation kit from Takara Bio (San Jose, CA, USA) was used following the manufacturer’s instructions. DNA isolation from the precipitate was performed using (1) a Wizard^®^ Genomic DNA Purification Kit (Promega, Madison, WI, USA) and (2) a Zymo Quick-DNA™ Miniprep Plus Kit from Zymo Research (Irvine, CA, USA) following the manufacturer’s instructions. The final volumes of all isolates were 20 µL and 1 µL from each isolate, and were measured using a NanoDrop spectrophotometer (Thermo Fisher Scientific, Waltham, MA, USA), an Agilent High Sensitivity DNA Kit (Agilent Technologies, Santa Clara, CA, USA), and Qubit (Thermo Fisher Scientific, Waltham, MA, USA). To avoid bias from the extraction methods, two different DNA extraction protocols were applied to the supernatant and precipitate from the first ampule. The results indicated no differences between the methods. Therefore, for the second ampule, we only used the NucleoSpin cfDNA isolation kit from Takara Bio (San Jose, CA, USA) for the supernatant and the Zymo Quick-DNA™ Miniprep Plus Kit from Zymo Research (Irvine, CA, USA) for the precipitate.

### 2.2. Real-Time PCR

Real-time PCR reactions for detection of *H. influenzae*, *S. pyogenes*, and *S. pneumoniae* were performed by using Microbial DNA qPCR Assay Kits from Qiagen (Germantown, MD, USA) following the manufacturer’s instructions.

### 2.3. Library Construction and Sequencing

DNA from 4 isolations (2 supernatants and 2 precipitates) from the 1st ampule was pooled and processed through whole genome amplification (WGA) using the REPLI-g Advanced DNA Single Cell Kit for whole genome amplification (Qiagen, Germantown, MD, USA) following the manufacturer’s instructions. An Illumina sequencing library was made from the isolated DNA using a ThruPLEX DNA-Seq Kit from Takara Bio USA (San Jose, CA, USA) following the manufacturer’s instructions. The final sequencing library was analyzed with an Agilent 2100 Bioanalyzer using the Agilent High Sensitivity DNA Kit (Agilent Technologies, Santa Clara, CA, USA). The constructed library was diluted following Illumina sequencing standard protocols and sequenced on MiSeq and NextSeq 500 sequencers (Illumina, San Diego, CA, USA). A final library concentration of 10 pM was used with MiSeq Reagent Kits v2 on the MiSeq machine. A final library concentration of 1.8 pM was used with NextSeq 500 Kits v2 on the NextSeq machine. DNA from 2 isolations (1 supernatant and 1 precipitate) from the 2nd ampule of the vaccine was processed with the same WGA and library-making procedures. The negative control library was made by utilizing an equivalent volume of nucleotide-free dH_2_O as input, following the same procedures for WGA and library construction.

### 2.4. Data Analysis

Illumina reads were trimmed using Trimmomatic (version 0.39) [22] and mapped to reference genomes including *H. influenzae* (GCA_000931575.1), *S. pneumoniae* (GCA_002076835.1), and *S. pyogenes* (GCA_900475035.1) and *E. faecium* (GCA_009734005.2), using Bowtie2 (version 2.3.4.1) [23]. Subsequently, the reads were also mapped to a broader set of available complete genomes downloaded from NCBI, comprising 117 *H. influenzae*, 223 *S. pneumoniae*, 296 *S. pyogenes*, and 303 *E. faecium* genomes. Bacterial genome classification in all the samples was performed using Kraken2 (version 2.0.9) [24], utilizing its nt database on the trimmed, filtered, and collapsed reads. A consensus nucleotide sequence was generated with SAMtools (version 1.16) [25] with default settings. A reported SNP call using VarScan2 (version 2.4.3) [26] was the one that satisfied the following criteria at the SNP position: (1) there were more than 100 reads at that position, (2) the minimum base Phred quality score was 25, (3) the different bases were more than 10% of the aligned reads, (4) it passed the VarScan2 Strand Filter, and (5) the VarScan2 SNP call *p*-value was less than 0.05. Two methods were used to identify antibiotic resistance genes from the consensus bacterial genome obtained: (1) The consensus genomes were uploaded to the Comprehensive Antibiotic Resistance Database (CARD; card.mcmaster.ca) site, and a Resistance Gene Identifier (RGI) was used to identify potential antibiotic resistance genes. (2) The MEGARes V3.0 database containing sequence data for nearly 9000 hand-curated resistance genes for antimicrobial drugs, biocides, and metals [27] was downloaded on 24 August 2024 and MegaBLAST (version 2.5.0) [28] at NCBI was used to search the obtained consensus genomes of *H. influenzae*, *S. pyogenes*, and *E. faecium* against the downloaded database to identify highly similar sequences. The serotype of the generated consensus *H. influenzae* genome was predicted using the hicap program (version 1.0.4) [29] with default settings. The sequence type of the generated consensus *S. pyogenes* genome was determined by blastn searching the genome sequence against an M-type-specific trimmed DNA database downloaded on 11 September 2024 from the Centers for Disease Control and Prevention (CDC) *Streptococcus* Laboratory website (https://ftp.cdc.gov/pub/infectious_diseases/biotech/tsemm/, accessed on 29 September 2024). The Multilocus Sequence Typing (MLST) of the obtained *E. faecium* genome was performed at the PubMLST website (https://pubmlst.org/organisms/enterococcus-faecium, accessed on 29 September 2024) [30] using 8 BezdicekMLST genes [31].

### 2.5. Phylogenetic Trees

The Codon Tree pipeline at BV-BRC (https://www.bv-brc.org/, accessed on 1 August 2024) [32] was used to generate bacterial phylogenetic trees including the generated consensus genome sequence and the identified best-aligned genomes. First, the obtained consensus genomes were annotated using their bacteria annotation pipeline and then put through the Codon Tree pipeline with other input corresponding genomes. Briefly, the pipeline used the amino acid and nucleotide sequences from a defined number of the BV-BRC global Protein Families (PGFams) [33], which were picked randomly, to build an alignment and then generate a tree based on the differences within those selected sequences. Protein sequences were aligned using MUSCLE [34], and the nucleotide coding gene sequences were aligned using the Codon_align function of BioPython [35]. A concatenated alignment of all proteins and nucleotides was written to a PHYLIP-formatted file, and then a partition file for RaxML [36] was generated, describing the alignment in terms of the proteins and then the first, second, and third codon positions. Support values were generated using 100 rounds of the “Rapid” bootstrapping option [37] of RaxML, and Midpoint rooting was the default.

## 3. Results

### 3.1. DNA Recovery

**Figure 1:**
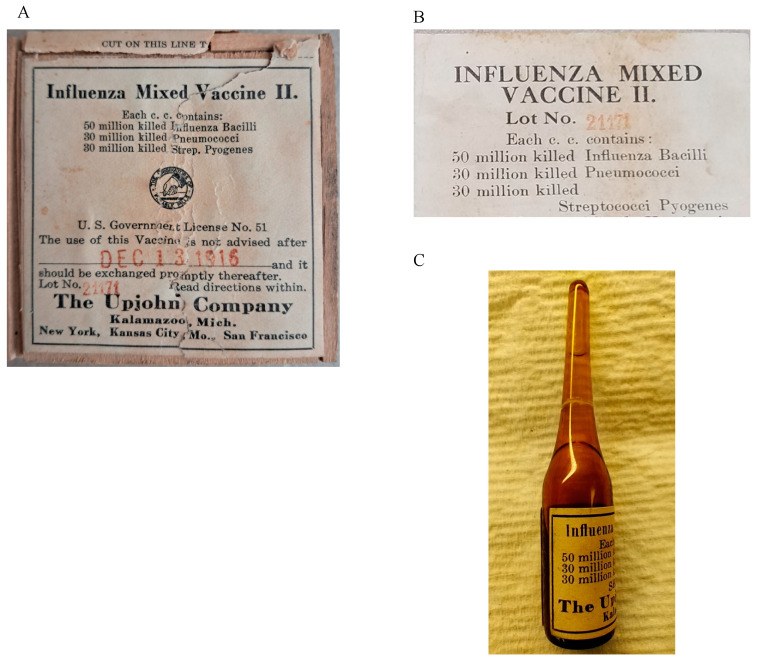
The bacteria vaccine material used in this study. (**A**) The label on the vaccine box. (**B**) The insert in the vaccine box. (**C**) The vaccine ampule.

Three unopened glass ampules of the bacterial vaccine, labeled “Influenza Mixed Vaccine II” and made by The Upjohn Company, Kalamazoo, Michigan, were provided by the Library of Congress from their collection (Figure 1). The Upjohn Company, one of the earliest pharmaceutical companies, was founded in 1884 by a physician, Dr. William Upjohn. It was among the first to produce a pill form of quinine. The company moved to Kalamazoo, Michigan, in 1885 (https://upjohn.net/corporate/early/early.htm, accessed on 29 September 2024). The ampules were labeled as Lot 21171, with an expiration date of 13 December 1916 (Figure 1A), and each CC was listed to contain 50 million colony-forming units (CFUs) of killed Influenza Bacilli, 30 million killed *Pneumococci*, and 30 million killed *Streptococci pyogenes* (Figure 1B). The manufacture date of the vaccine was not included on the label. In the vaccine ampules, there were visible small particles floating in the liquid or settled at the bottom. After the first ampule was opened, the measured total volume of the vaccine solution was ~1100 µL. DNA extractions were performed twice with different methods on both solid and liquid portions from ~500 µL of the vaccine contents. However, no detectable DNA concentrations were observed using the NanoDrop (Thermo Fisher Scientific, Waltham, MA, USA), Bioanalyzer High Sensitivity Chip (Agilent Technologies, Santa Clara, CA, USA), or Qubit (Thermo Fisher Scientific, Waltham, MA, USA) techniques.

Real-time PCR of the 16S rRNA gene was performed to detect *Haemophilus influenzae* (*H. influenzae*, labeled as Influenza Bacilli on the vaccine ampule), *Streptococcus pneumoniae* (*S. pneumoniae*, labeled as Pneumococci on the vaccine), and *Streptococcus pyogenes* (*S. pyogenes*, labeled as Streptococci Pyogenes on the vaccine) in DNA extractions from both the supernatant and precipitation fractions of the vaccine. The results of the 16S rRNA gene real-time PCRs are shown in Table 1 and confirmed the following: (1) The real-time PCR reactions worked well, as demonstrated by the results of the negative and positive controls; (2) in the precipitate, the *H. influenzae* 16S rRNA gene was detected, with a Ct value of 36.133, and *S. pyogenes* was also detected, but with a Ct value of 39.915; (3) only *H. influenzae* DNA was detected in the supernatant, with a Ct value of 39.738. DNA from four isolations (two supernatants and two precipitates) from the first vial was pooled together and subjected to whole genome amplification (WGA) using the REPLI-g Advanced DNA Single Cell Kit (Qiagen, Germantown, MD, USA). The DNA profile obtained from this amplification is presented in Supplemental Figure S1.

DNA isolations and WGA from the second opened vaccine ampule were the same as above, except only two DNA isolations were performed (one supernatant and one precipitate). To conserve the amount of DNA obtained, 16S rRNA gene real-time PCR was only performed on *H. influenzae* for the second vaccine ampule, and the results were similar to those from the first ampule (Supplemental Table S1).

These findings indicated that the DNA yield from the supernatant was significantly lower than that from the precipitate, and no DNA from *S. pneumoniae* was detected in any samples. The WGA significantly increased the total DNA amount obtained.

### 3.2. Illumina Sequencing

The amplified DNA from the pooled vaccine material was used to make Illumina libraries, which were sequenced on a MiSeq and NextSeq 500 sequencer. A negative control library was made side-by-side with the processing of the second ampule of vaccine using nucleotide-free dH_2_O as input, following identical procedures for WGA and library construction. Supplemental Table S2 shows the sequencing data generated from DNA libraries of the two vaccine vials on MiSeq and NextSeq, respectively. All sequences obtained have been deposited as a series into the GenBank SRA database (accession no. PRJNA1220618).

### 3.3. Sequence Alignment to the Three Bacterial Reference Genomes

As shown in Figure 1B, this Influenza Mixed Vaccine II was labeled as containing 50 million killed Influenza Bacilli (*H. influenzae*), 30 million killed Pneumococci (*S. pneumoniae*), and 30 million killed Streptococci Pyogenes (*S. pyogenes*). Therefore, reference genomes of *H. influenzae* (GCA_000931575.1), *S. pneumoniae* (GCA_002076835.1), and *S. pyogenes* (GCA_900475035.1) were downloaded from NCBI. All generated Illumina reads from the first ampule of the vaccine were trimmed and aligned to these reference genomes using Bowtie2 [23], and the results are shown in Table 1. Sequence alignment results from both MiSeq and NextSeq data, including overall alignment rate (the percentage of aligned reads mapped onto the reference genomes out of the total trimmed reads) and genome coverage rate (the portion of the reference genomes aligned by reads), were analyzed. These results confirmed that *H. influenzae* had the highest DNA content, while *S. pneumoniae* had the lowest, consistent with real-time PCR findings (Supplemental Table S1). Additionally, the higher number of reads from the NextSeq platform significantly improved reference genome coverage, especially for *H. influenzae* (93.2%) and *S. pyogenes* (53.8%), for the first ampule (Table 1).

Sequences generated from the second ampule of the vaccine and from the negative control were aligned to the same three reference genomes, and the results are also shown in Table 1. However, the alignment results showed that the relative amounts of the three vaccine bacteria in the second ampule were quite different from those in the first ampule. While the first ampule contained DNA predominantly from *H. influenzae*, followed by *S. pyogenes*, and nearly none from *S. pneumoniae*, the second ampule showed DNA from *S. pyogenes* as the most abundant, followed by *H. influenzae*, with similarly negligible amounts of *S. pneumoniae*. Although the percentage of aligned reads of *S. pneumoniae* in the second ampule was higher than that of *H. influenzae*, the genome coverage rate of the *S. pneumoniae* reference genome was only 0.046 and was much lower than the 0.499 from *H. influenzae*. When analyzed, 90.52% of the reads aligned to *S. pneumoniae* were also aligned to the *S. pyogenes* reference genome, which likely indicates that these reads are conserved between *S. pneumoniae* and *S. pyogenes* genomes.

Alignment results from the negative control experiment show that only tens to hundreds of reads aligned to the reference genomes, compared to tens to hundreds of thousands of reads aligned from the vaccine experimental groups. This demonstrated that there was no significant contamination by these bacteria during our experimental processing.

### 3.4. Identifying the Best-Aligned Species from the H. influenzae, S. pneumoniae, and S. pyogenes Genomes

To identify the most well-matched strains of *H. influenzae, S. pneumoniae,* and *S. pyogenes* in this vaccine, all sequences were aligned to 117 *H. influenzae*, 223 *S. pneumoniae*, and 296 *S. pyogenes* complete genomes, respectively, which were downloaded from NCBI on 7 June 2024. The numbers of aligned reads and the rates of genome coverage are shown in Supplemental Tables S3 (*H. influenzae*), S4 (*S. pneumoniae*), and S5 (*S. pyogenes*). The top aligned *H. influenzae* genome was CP085949.1, *H. influenzae* strain FDAARGOS_1562 (GCA_020735825.1), with a chromosome coverage rate of 99.5% from the first ampule (Supplemental Table S3). The top aligned *S. pneumoniae* genome was CP035260.1, *S. pneumoniae* strain TVO_1901925, with a chromosome coverage rate of only 7.1% from the first ampule (Supplemental Table S4). The top aligned *S. pyogenes* genome was AP014585.1, *S. pyogenes* strain MTB314 (GCA_001547835.1), with a chromosome coverage rate of 95.7% from the second ampule (Supplemental Table S5). Therefore, although the two bacterial vaccine ampules were labeled as containing the same amounts of killed *H. influenzae*, *S. pneumoniae*, and *S. pyogenes*, sequence alignments of the data from them revealed that they contained little detectable *S. pneumoniae* DNA. The first ampule mainly contained *H. influenzae* DNA, while the second vial mainly contained *S. pyogenes* DNA.

### 3.5. Metagenomic Analyses

**Figure 2:**
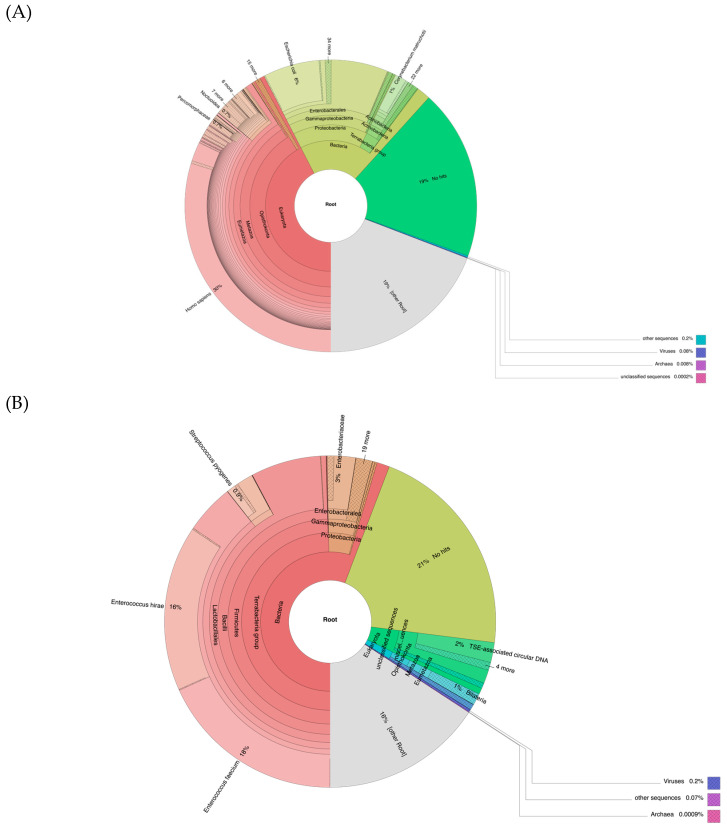

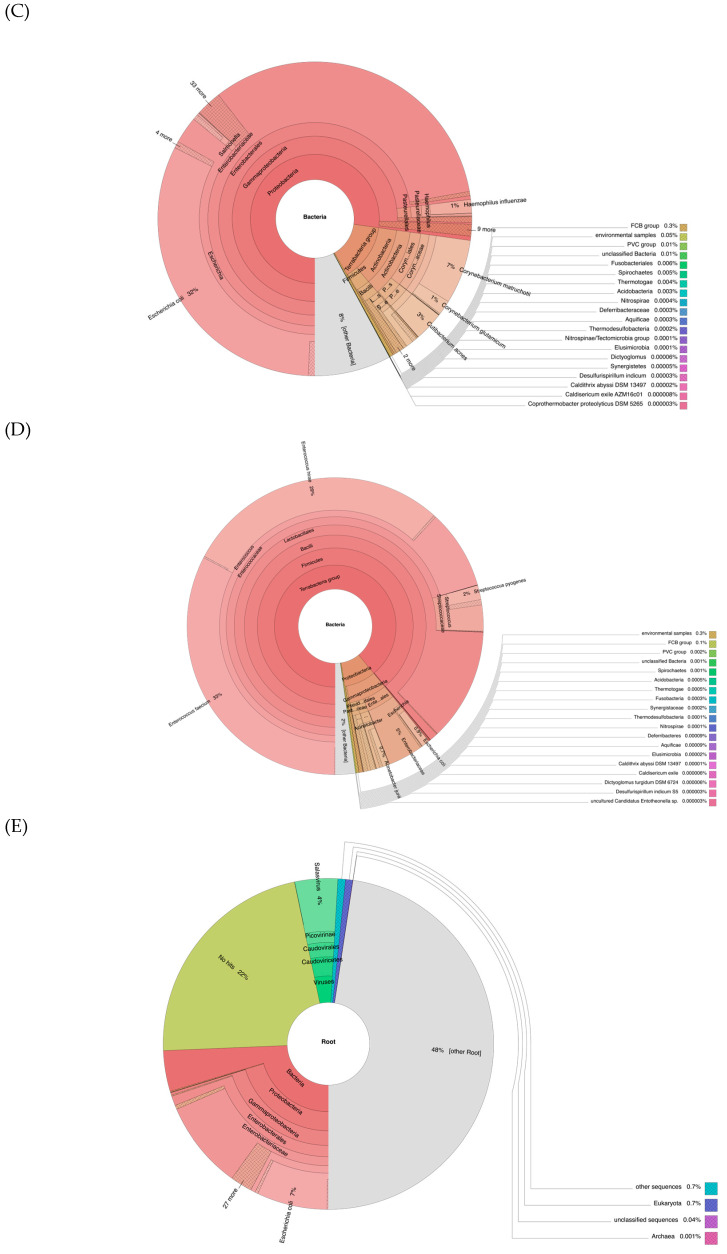

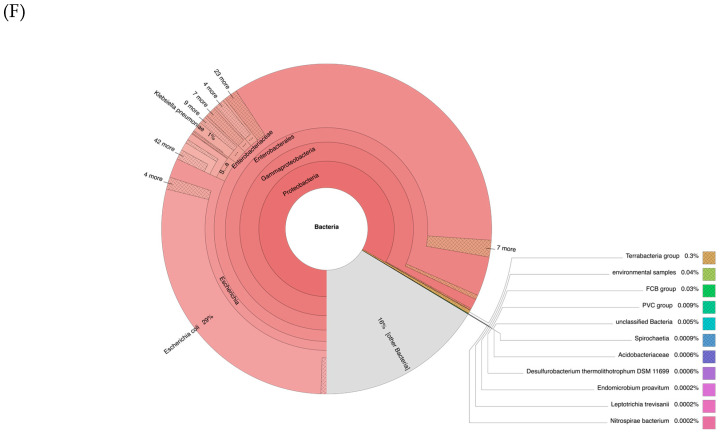
Metagenomics analysis of NextSeq data. (**A**): Data from the 1st ampule at the root level. (**B**): Data from the 2nd ampule at the root level. (**C**): Data from the 1st ampule at the bacterial level. (**D**): Data from the 2nd ampule at the bacterial level. (**E**): Data from the negative control at the root level. (**F**): Data from the negative control at the bacterial level.

Metagenomic analyses were performed on trimmed, filtered, and collapsed reads from the MiSeq and NextSeq runs of DNA from the two ampules using Kraken2 [24]. The results from the NextSeq data are shown in Figure 2 while the results from the MiSeq data were remarkably similar and are shown in Supplemental Figure S2.

From the data of the first ampule, at the root level (Figure 2A), the most classified reads were *Homo sapiens*, and the most classified reads at the bacterial level were *E. coli* (6% at the root (Figure 2A) and 32% at the bacterial level (Figure 2C)). They also showed 1% of reads classified as *H. influenzae*, 0.07% of reads classified as *S. pyogenes*, and 0.01% of reads classified as *S. pneumoniae* at the bacterial level, consistent with the real-time PCR and sequencing results for the amounts of these three labeled bacteria. From the data of the second ampule, the most classified reads were *Enterococcus faecium* (*E. faecium*) at the root level (18%) (Figure 2B) and at the bacteria level (33%) (Figure 2D). They also showed 0.5% of reads classified as *H. influenzae*, 2% of reads classified as *S. pyogenes*, and 0.002% of reads classified as *S. pneumoniae* at the bacterial level (Figure 2D), which is also in agreement with their real-time PCR and sequencing results. Lastly, from the negative control data, the most classified reads at the root level belong to *Escherichia coli* (*E. coli*) (7% at the root level (Figure 2E), and 29% at the bacterial level (Figure 2F)). There were no reads classified as *H. influenzae*, *S. pneumoniae*, or *S. pyogenes* in the negative control library data.

### 3.6. Sequence Alignment to Enterococcus faecium from the Data of the Second Ampule

Based on metagenomic analysis, most of the bacterial DNA revealed in the second ampule of the vaccine matched *E. faecium* (Figure 2D). The reference genome of *E. faecium* (strain SRR24, GCA_009734005.2) was downloaded from NCBI. All generated Illumina reads from the second ampule were trimmed and aligned to this genome using Bowtie2 [23]. Among the total of 431,906,108 trimmed reads, 52,172,022 reads (12.08%) were aligned to the *E. faecium* genome (49,112,468 (11.37%) aligned exactly one time and 3,059,554 (0.71%) aligned > one time), which covered 82.7% of the *E. faecium* genome, with an average genome coverage of 1025.54 times. To identify the best-matched strain of *E. faecium* in this vaccine, all sequences were aligned to all 303 complete genomes of *E. faecium* that were downloaded on 17 July 2024. The numbers of aligned reads and the rates of genome coverage are shown in Supplemental Table S4. The top aligned *E. faecium* genome was CP021885.1 of *E. faecium* strain WEFA23 (GCA_002850515.1), with a chromosome coverage rate of 97.4% (Supplemental Table S6). Therefore, the second ampule of the vaccine also contained the whole genome of *E. faecium*.

### 3.7. Consensus Sequences of H. influenzae, S. pyogenes, and E. faecium in Vaccine

**Figure 3:**
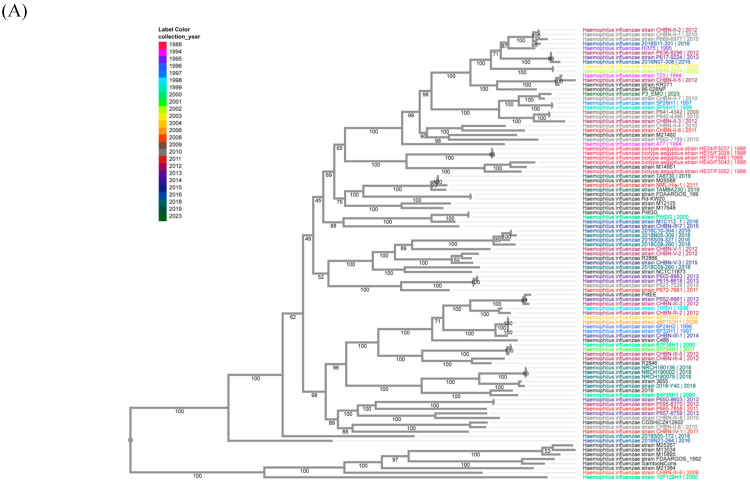

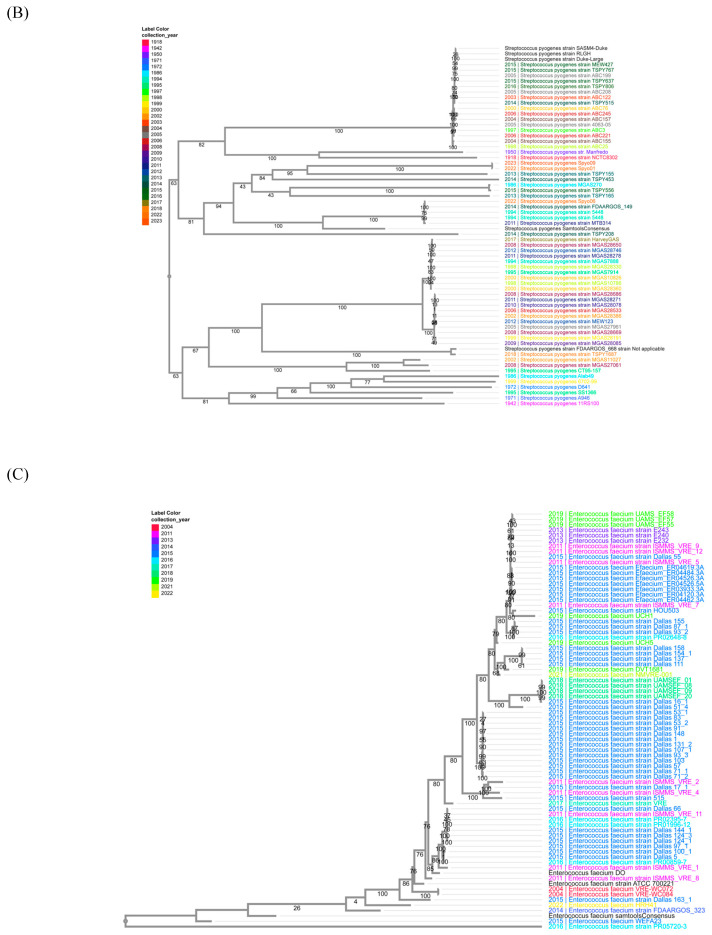
Phylogenetic codon tree of generated consensus genomes with their corresponding complete genomes with their collection years. (**A**): *H. influenzae* codon tree. (**B**): *S. pyogenes* codon tree. (**C**): *E. faecium* codon tree. The collection years for each genome, if available, are also labeled with colors.

Consensus sequences of *H. influenzae*, *S. pyogenes*, and *E. faecium* in the sequenced vaccine were generated based on the reference genomes with the highest genome coverages. All these consensus genomes were submitted to GenBank, with accession numbers of CP185964 for *H. influenzae*, CP185963 for *S. pyogenes*, and CP185962 for *E. faecium*. Totals of 935, 905, and 11,873 single-nucleotide polymorphisms (SNPs) were identified in the consensus genomes of *H. influenzae*, *S. pyogenes*, and *E. faecium*, respectively, compared to their aligned reference genomes (Supplemental Tables S7–S9). The generated consensus genome sequences of *H. influenzae*, *S. pyogenes*, and *E. faecium* were compared to their corresponding complete available genomes and constructed phylogenetic trees at the BV-BRC website (https://www.bv-brc.org/, accessed on 1 August 2024) using their Codon Tree pipeline [32]. The *H. influenzae* phylogenetic tree including the generated consensus genome sequence, and its best-aligned reference, strain FDAARGOS_1562 (GCA_020735825.1), and the 97 complete human *H. influenzae* genomes at BC_BRC is shown in Figure 3A. The *S. pyogenes* phylogenetic tree including the generated consensus genome sequence, and its best-aligned reference, strain MTB314 (GCA_001547835.1), and the 64 complete North American *S. pyogenes* genomes at BC_BRC is shown in Figure 3B. The *E. faecium* phylogenetic tree including the generated consensus genome sequence, and its best-aligned reference, strain WEFA23 (GCA_002850515.1), and the 76 complete North American human *E. faecium* genomes at BC_BRC is shown in Figure 3C. Within the constructed phylogenetic trees, the earliest collection years for genomes are 1988 for *H. influenzae* strain HE7/F1946, 1918 for *S. pyogenes* strain NCTC8302, and 2004 for *E. faecium* strain VRE-WC072. These three obtained consensus genomes of *H. influenzae*, *S. pyogenes*, and *E. faecium* are closest to their best-aligned genomes, respectively, rather than the genomes with the earliest collection years (Figure 3).

The serotype of the obtained consensus *H. influenzae* genome was assessed with hicap, a program that can perform serotyping of the *H. influenzae* capsule locus in silico based on genome sequences [29], and it determined that this obtained genome from the 1916 bacterial vaccine is *H. influenzae* type-f (Supplemental Table S10). The serotype of the obtained consensus *S. pyogenes* genome was determined by comparing both the genome sequence and the identified *emm* gene sequence (based on the annotated reference genome) against the *S. pyogenes* M-type-specific trimmed DNA database downloaded from the CDC (https://ftp.cdc.gov/pub/infectious_diseases/biotech/tsemm/, accessed on 11 September 2024). The top hit from the database was emm1.149, indicating that the *S. pyogenes* genome recovered from the ampule belongs to the emm1.149 serotype. Typing of the consensus *E. faecium* genome was performed using the PubMLST database (https://pubmlst.org, accessed on 29 September 2024) and the MLST gene list [31,38]. Seven of eight MLST loci were identified, as shown in Supplemental Table S11. The profile appears to represent a novel sequence type (ST), as it did not match any existing ST in the *E. faecium* PubMLST database.

In addition, the antimicrobial resistance (AMR) profiles of these three generated bacterial consensus genomes were evaluated. First, the Resistance Gene Identifier (RGI) tool from the Comprehensive Antibiotic Resistance Database (CARD; card.mcmaster.ca) was used to identify potential antibiotic resistance genes (ARGs) [39]. No ARGs were identified in the *H. influenzae* consensus genome. However, two ARGs were detected in the *S. pyogenes* consensus genome, and four ARGs were identified in the *E. faecium* consensus genome, based on the stringent criteria set by the RGI tool (Table 2).

Secondly, MegaBLAST [28] was used to identify highly similar sequences from the MEGARes V3.0 database, which contains nearly 9000 hand-curated resistance genes related to antimicrobial drugs, biocides, and metals [27]. The search results revealed that 426 regions of the *H. influenzae* consensus genome matched 90 resistance genes (Supplemental Table S12), 436 regions of the *S. pyogenes* consensus genome matched 67 resistance genes (Supplemental Table S13), and 482 regions of the *E. faecium* consensus genome matched 95 resistance genes (Supplemental Table S14). Thus, all three bacterial strains obtained from the 1916 bacterial vaccine contained potential AMR genes.

## 4. Discussion

The bacterial vaccine analyzed in this study was produced by The Upjohn Company in Kalamazoo, Michigan [40]. However, we were unable to find further information about the bacterial vaccines made by this company around 1916. Given the era, it is likely that the production and testing protocols for these vaccines were neither standardized nor scientifically monitored. For example, during the 1918 influenza pandemic, with the death toll rising dramatically, scientists and physicians relentlessly pursued the pathogen and potential cures, resulting in the rapid development of numerous experimental or “boutique” bacterial vaccines. At one point, Illinois had 18 different bacterial vaccines produced [41]. Modern genetic analyses have revealed that some of these historical vaccines were not what they were believed to be. For example, sequencing of a historical 1902 smallpox vaccine unexpectedly revealed horsepox virus DNA instead of vaccinia virus [42,43]. Similarly, our previous study of a 1944 Rocky Mountain spotted fever (RMSF) vaccine found substantial DNA from non-target organisms, including *Dermacentor andersoni* (the tick vector of RMSF) and *Coxiella burnetii*, and the pathogen responsible for Q-fever, rather than solely *Rickettsia rickettsii*, the causative agent of RMSF [44].

Based on the DNA genome length and weight relationship [45] and the total millions of CFUs of killed bacteria in the ampules as labeled, the total amount of DNA in the ampules should theoretically be about 200 ng, which should be measurable by the quantification methods used. The undetectable DNA amount recovered from both vaccine ampules examined here could thus be a result of the degradation of bacterial DNA during storage under ambient conditions for more than a century. However, the absence of *S. pneumoniae* DNA, despite its being listed on the labels of both ampules (Figure 1), in both sequenced libraries of the 1916 bacterial vaccine is likely due to production errors rather than DNA degradation, given the detection of other bacterial full genomes. Moreover, the discrepancies between the two ampules, despite sharing the same lot number, further underscore likely inconsistent production procedures at that time. In addition, the substantial proportion of human reads detected in the first ampule (Figure 2A) indicates that the vaccine material most likely originated from a human-associated source, such as a throat swab or sputum sample, which was common practice in the earlier 1900s [9]. Therefore, these findings highlight that historical vaccines may not have contained the correct or intended pathogens, raising questions about their effectiveness and safety by modern standards.

### 4.1. Serotypes of Consensus Genomes Obtained

*H. influenzae* was first described in 1893 by Richard Pfeiffer during the 1889–1890 influenza pandemic, and he mistakenly concluded that it was the causative agent of influenza [6]. During the beginning stages of the 1918 influenza pandemic, *H. influenzae* continued to be regarded as the causative pathogen, leading to the development of vaccines based on this assumption [46]. The serotypes of *H. influenzae* were unknown until 1931, when they were first described by Pittman [47]. These serotypes have been classified based on differences in their capsular polysaccharide, with serotypes ranging from a to f, as well as non-typeable strains. Prior to the introduction of effective vaccination (Hib type), *H. influenzae* serotype b (Hib) was the most common serotype causing invasive disease, and it was a leading cause of meningitis and epiglottitis in children [48,49]. Given that the bacterial strains used in vaccines during the 1918 influenza pandemic were derived from patient swab samples, the *H. influenzae* genome analyzed in this study likely represents strains/serotypes that were circulating in 1916, even during the 1918 pandemic.

Serotyping of *Streptococcus pyogenes* was first introduced in 1928 by Lancefield, utilizing antigen–antibody reactions with type-specific antisera to identify the M protein on the surface of Group A Streptococci [50]. Later, *emm* typing became the preferred method, which is based on sequence analysis of the N-terminal hypervariable region of the M protein gene [51,52]. This technique correlates almost perfectly with the results of M serotyping [53]. Currently, the *emm*-typing scheme has identified 270 different *emm*-types at the CDC *Streptococcus* Laboratory website [54] (https://ftp.cdc.gov/pub/infectious_diseases/biotech/tsemm/). The obtained *S. pyogenes* genome in the current study has the strongest match to *emm*1.149 based on the CDC *emm*-type database. The *emm*1 type of *S. pyogenes* is the top 1 among the 70 most common *emm*-types as a proportion of worldwide population-adapted isolates and belongs to the 15 vaccine representative isolates [55]. The diseases that are caused by the *emm*1 type of *S. pyogenes* include pharyngitis, scarlet fever, acute rheumatic fever, acute post-streptococcal glomerulonephritis, bacteremia, puerperal sepsis, puerperal sepsis, and streptococcal toxic shock syndrome [56], and it is one of the throat-associated *emm*-types frequently recovered from most countries [55]. The *S. pyogenes* recovered in this 1916 vaccine might be the oldest identified *emm*1 type of the *S. pyogenes* strain so far.

*Enterococcus faecium* is one of the most important pathogens causing health care-associated infections, and its clinically related sequence types include ST17, ST18, ST25, ST78, ST112, ST117, ST296, ST473, and ST474 [57,58,59]. The sequence type of the *E. faecium* genome obtained for this study was determined at the PubMLST website (https://pubmlst.org/organisms/enterococcus-faecium) [30] using the newly incorporated BezdicekMLST scheme which targets eight genes [31]. The consensus *E. faecium* genome from this vaccine matched seven of the eight target genes (Supplemental Table S11). However, no exact sequence type match for *E. faecium* is present in the database. The closest match found among a total of 1190 sequence types in the database was ST422, and its profile is shown in Table 3. The 1916 vaccine *E. faecium* genome obtained here matches six loci (copA, dnaE, HP2027, mdlA, narB, and uvrA) of ST422, but differs at the rpoD locus and lacks pbp2B (Supplemental Table S11). A query of the obtained *E. faecium* genome for pbp2B exclusively at PubMLST revealed the closest match: pbp2B is profile 80 with two base differences. Therefore, the sequence type of this obtained *E. faecium* genome from the 1916 bacterial vaccine is likely a new sequence type.

### 4.2. Antimicrobial Resistance (AMR) Profiles in Obtained Bacterial Strains

When evaluating the antimicrobial resistance (AMR) profiles of the three obtained bacterial genomes, the Resistance Gene Identifier (RGI) tool from the Comprehensive Antibiotic Resistance Database (CARD; card.mcmaster.ca) [39] identified two antibiotic resistance genes (ARGs) in *S. pyogenes* and four ARGs in *E. faecium*, but none in the *H. influenzae* genome. The *S. pyogenes* genome harbors PatA and B homologs, which constitute a heterodimeric ABC-transporter, which can confer clinically relevant levels of fluoriquinolone resistance if overexpressed [60,61]. VanY homologs were detected in the *S. pyogenes* and *E. faecium* genomes, with low nucleotide identity. It is unlikely that these homologs confer glycopeptide resistance on their own [62], even if functional as a D,D-carboxypeptidase. The *E. faecium* strain WEFA23, which is the closest relative to the *E. faecium* vaccine isolate, also harbors the VanY homologs and is susceptible to vancomycin [63]. Furthermore, aac(6′)-Ii was identified in the *E. faecium* genome, which encodes a 6′-N-aminoglycoside acetyltransferase that confers intrinsic, low-level resistance to aminoglycosides [64], including amikacin and kanamycin. The major facilitator superfamily efflux pump EfmA is chromosomally encoded in *E. faecium* and may confer low-level resistance to several antibiotics, including macrolides and fluoroquinolones [65]. Altogether, the RGI tool identified genes involved in low-level, intrinsic antibiotic resistance against fluoroquinolones, aminoglycosides, and macrolides.

When these genomes were compared against the MEGARes V3.0 database [27], a significantly larger number of potential resistance genes was identified in all three genomes (Supplemental Tables S8–S10). The discrepancy between the number of ARGs identified by RGI and MEGARes is likely because of the RGI tool’s stricter screening criteria and its use of a smaller database. Additionally, many of the hits from the MEGARes database still require single-nucleotide polymorphism (SNP) confirmation. The genomes of the vaccine components date from the early 20th century. The loci in the MEGARes database are divergent from the ancestral genomes of the vaccine components, and therefore many loci in resistance-associated genes were flagged as putatively involved in AMR, although they may simply represent the ancestral state of the locus, rather than a resistance mutation. Similar to RGI, MEGARes also identified several efflux pumps, which can confer clinically relevant levels of AMR. However, achieving minimal inhibitory concentrations beyond the therapeutic window of a drug by efflux often requires mutational changes, e.g., in transcriptional repressors, that cause the overexpression of the efflux pumps [66].

Nevertheless, it is well established that antibiotic resistance in bacteria is an ancient and naturally occurring phenomenon. Metagenomic analyses of rigorously authenticated ancient DNA from 30,000-year-old Beringian permafrost sediments identified a highly diverse collection of genes encoding resistance to β-lactam, tetracycline, and glycopeptide antibiotics, and structure and function studies on the complete vancomycin resistance element VanA showed its similarity to modern enzymes of VanA from vancomycin-resistant *E. faecium* [67]. In addition, bacteria isolated from Lechuguilla Cave in New Mexico, which has been isolated for over 4 million years, demonstrated high resistance to many commercially available antibiotics [68]. In a recent study of a fatal 1918 influenza case, antibiotic resistance genes were also identified in the *Rhodococcus* genome obtained from postmortem formalin-fixed, paraffin-embedded (FFPE) lung tissue [69]. These findings, along with the current study, further support the concept that antibiotic resistance predates human antibiotic use and has long been a part of natural microbial evolution.

Lastly, preventing DNA contamination throughout the experimental process was essential; therefore, we performed negative control experimental sampling and processing alongside the experimental processing on the second ampule vial. According to the document of the REPLI-g Advanced DNA Single Cell Kit for whole genome amplification (Qiagen, Germantown, MD, USA), up to 20 μg of DNA can be present in negative (no-template) controls because DNA is generated during the REPLI-g Advanced Single Cell reaction by random extension of primer-dimers, generating a high-molecular-weight product. This DNA does not affect the quality of the actual samples and does not give a positive result in downstream assays. Therefore, a no-template control was conducted alongside the DNA sample isolated from the second vaccine vial. Comparative analysis of sequence reads from both the no-template control and the DNA from the second vaccine ampule revealed that only *E. coli* reads were identified in the no-template control, with none detected in the sample from the second ampule. On the other hand, most of the bacterial DNA identified in the first vaccine ampule was from *E. coli*. However, because there was no negative control during the analysis of the first vial, as it was part of the initial methodological investigation, we cannot definitively rule out the possibility that the *E. coli* reads resulted from contamination during the process. In addition, the handling of vaccine vials and sample processing were consistently conducted in biological safety cabinets. Furthermore, the laboratory where the sample processing and sequencing occurred, as well as other laboratories in the building, has no history of prior research involving these bacteria.

### 4.3. Limitations

This study has several limitations. First, the total DNA amount recovered from the century-old vaccine ampules was very low, which meant that whole genome amplification was required to further perform genetic analysis. However, whole genome amplification may introduce bias [70], which could have affected the analysis. Second, the microbial content did not fully match the labeled composition, and the original vaccine preparation methods and storage conditions remain unknown, which could further limit our interpretation. Despite these limitations, our results provide valuable insights into early vaccine formulations and demonstrate the feasibility of applying modern molecular techniques to historical biomedical materials.

## 5. Conclusions

This study presents a genomic analysis of two unopened ampules of a mixed bacterial vaccine produced in 1916, during the early era of bacterial pneumonia vaccines, preceding the 1918 influenza pandemic. Using modern NGS technologies, we identified discrepancies between the labeled contents and the actual bacterial DNA recovered, including the unexpected presence of *Enterococcus faecium*. Despite the age, consensus genomes of *H. influenzae*, *S. pyogenes*, and *E. faecium* were successfully assembled and analyzed for serotype, phylogeny, and antibiotic resistance genes. These findings offer valuable insights into early 20th-century vaccine formulations and highlight both the promise and limitations of historical bacterial vaccine production. This work underscores the potential of applying modern molecular tools to archived biomedical materials to better understand past public health responses.

## Figures and Tables

**Table 1:** Reads aligned to the three bacterial reference genomes.

Vaccine Vial	Sequencer	Reference Genomes	Total Reads	Reads Aligned 1 Time	Reads Aligned > 1 Time	Overall Alignment Rate	Genome Coverage Rate
First ampule	MiSeq	*H. influenzae* (CP007470.1)	22,551,907	34,700	1696	0.14%	0.4916499
		*S. pneumoniae* (CP020549.1)	22,551,907	2256	34	0.01%	0.0173710
		*S. pyogenes* (LS483338.1)	22,551,907	14,483	1079	0.06%	0.0775702
	NextSeq	*H. influenzae* (CP007470.1)	517,835,296	665,650	27,100	0.13%	0.9324558
		*S. pneumoniae* (CP020549.1)	517,835,296	46,512	570	0.01%	0.0586087
		*S. pyogenes* (LS483338.1)	517,835,296	223,372	11,997	0.05%	0.5378864
Second ampule	MiSeq	*H. influenzae* (CP007470.1)	15,368,293	47,655	18,466	0.43%	0.0455131
		*S. pneumoniae* (CP020549.1)	15,368,293	78,295	76	0.51%	0.0115521
		*S. pyogenes* (LS483338.1)	15,368,293	144,894	76,102	1.44%	0.2916496
	NextSeq	*H. influenzae* (CP007470.1)	416,537,815	1,303,480	489,618	0.43%	0.4993817
		*S. pneumoniae* (CP020549.1)	416,537,815	2,119,681	2036	0.51%	0.0461191
		*S. pyogenes* (LS483338.1)	416,537,815	3,873,131	2,060,507	1.42%	0.9377816
Negative control	MiSeq	*H. influenzae* (CP007470.1)	10,199,050	45	612	0.01%	0.0007859
		*S. pneumoniae* (CP020549.1)	10,199,050	26	0	0.00%	0.0004585
		*S. pyogenes* (LS483338.1)	10,199,050	44	28	0.00%	0.0017173

**Table 2:** Antibiotic resistance genes identified with RGI.

Consensus Genomes	Start	Stop	Orientation	Model Type	Drug Class	Resistance Mechanism	AMR Gene Family
*H. influenzae*	N/A	N/A	N/A	N/A	N/A	N/A	N/A
*S. pyogenes*	202,711	204,495	+	protein homolog model	fluoroquinolone antibiotic	antibiotic efflux	ATP-binding cassette (ABC) antibiotic efflux pump
	1,370,493	1,371,236	-	protein homolog model	glycopeptide antibiotic	antibiotic target alteration	vanY; glycopeptide resistance gene cluster
*E. faecium*	99,816	100,640	-	protein homolog model	glycopeptide antibiotic	antibiotic target alteration	vanY; glycopeptide resistance gene cluster
	472,276	472,938	-	protein homolog model	glycopeptide antibiotic	antibiotic target alteration	vanY; glycopeptide resistance gene cluster
	630,791	631,339	-	protein homolog model	aminoglycoside antibiotic	antibiotic inactivation	AAC(6′)
	1,624,902	1,626,188	+	protein homolog model	macrolide antibiotic; fluoroquinolone antibiotic	antibiotic efflux	major facilitator superfamily (MFS) antibiotic efflux pump

**Table 3:** The allele profile of the closest sequence type to the obtained genome of *E. faecium* in PubMLST.

ST	copA	dnaE	HP2027	mdlA	narB	pbp2B	rpoD	uvrA	Clonal_Complex
422	6	3	3	9	3	3	4	2	CC89

## Data Availability

The datasets generated and/or analyzed during the current study are available in the NCBI database under the following accession numbers: PRJNA1220618 for NGS data, and CP185964, CP185963, and CP185962 for the consensus genomes of *H. influenzae*, *S. pyogenes*, and *E. faecium*, respectively.

## Supplementary Materials

The following supporting information can be downloaded at: https://www.mdpi.com/article/10.3390/vaccines13050491/s1. Figure S1: Amplified DNA profile from pooled isolations of vaccine measured using a High Sensitivity DNA chip on an Agilent Bioanalyzer; Figure S2: Metagenomics analysis of MiSeq data. Table S1: Real-time PCR of 16S rRNA gene of *Haemophilus influenzae*, *Streptococcus pneumoniae*, and *Streptococcus pyogenes*; Table S2: Generated sequence from 2 vaccine vials on MiSeq and NextSeq 500; Table S3: The alignment results of the 1st vial and the 2nd vial to available complete *H. influenzae* genomes; Table S4: The alignment results of the 1st vial and the 2nd vial to available complete *S. pneumoniae* genomes; Table S5: The alignment results of the 1st vial and the 2nd vial to available complete *S. pyogenes* genomes; Table S6: The alignment results of the 2nd vial to available complete *E. Faecium* genomes; Table S7: The detected SNPs from consensus *H. influenzae* genome; Table S8: The detected SNPs from consensus *S. pyogenes* genome; Table S9: The detected SNPs from consensus *E. faecium* genome; Table S10: Serotype of obtained *H. influenzae* genome by hicap; Table S11: MLST typing of *E. faecium* genome at PubMLST at using 8 MLST genes [31]; Table S12: The *H. influenzae* consensus genome blast hits of resistance genes related to antimicrobial drugs, biocides, and metals from MEGARes V3.0 database; Table S13: The *S. spyogenes* consensus genome blast hits of resistance genes related to antimicrobial drugs, biocides, and metals from MEGARes V3.0 database; Table S14: The *E. faecium* consensus genome blast hits of resistance genes related to antimicrobial drugs, biocides, and metals from MEGARes V3.0 database.
