# Supplementary material for: Genetic Sequencing of a Bacterial Pneumonia Vaccine Produced in 1916
Source: Vaccines (Basel). 2025 May 2;13(5):491. doi: 10.3390/vaccines13050491 (PMC12115763; doi:10.3390/vaccines13050491)
Supplement: Supplementary file 1 [file vaccines-13-00491-s001.zip › SupplementalFigureS2_MiSeqMeta.pdf]

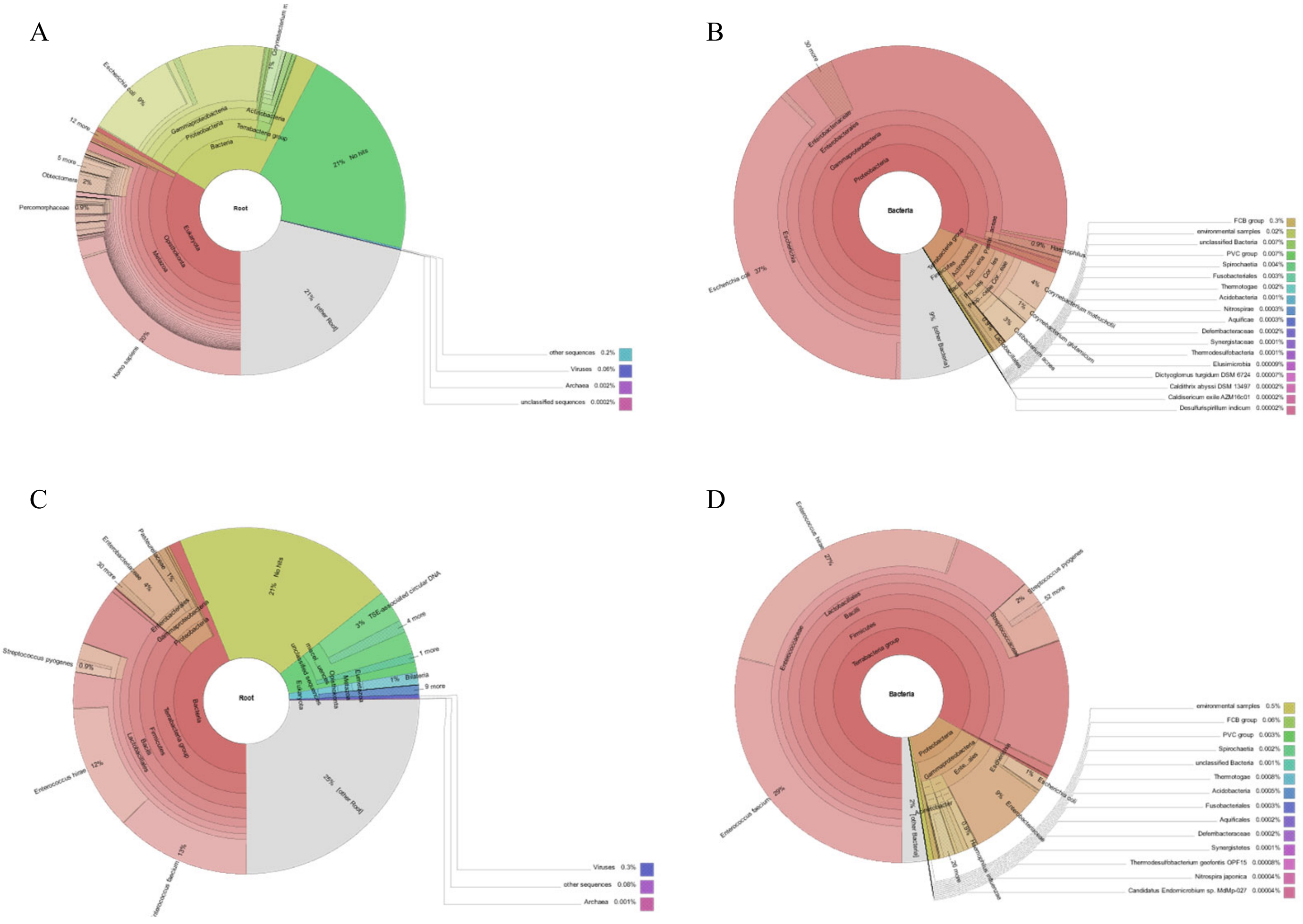

Supplemental Figure S2. Metagenomics analysis of MiSeq data. A: Paired-end data from the 1st ampule at root level. B: Paired-end data from the 1st ampule at bacterial level. C: Data from the 2nd ampule at root level. D. Data from the 2nd ampule at bacterial level.
