# Supplementary material for: Genetic Sequencing of a Bacterial Pneumonia Vaccine Produced in 1916
Source: Vaccines (Basel). 2025 May 2;13(5):491. doi: 10.3390/vaccines13050491 (PMC12115763; doi:10.3390/vaccines13050491)
Supplement: Supplementary file 1 [file vaccines-13-00491-s001.zip › SupplementalTableS10_Hicap.pdf]

Supplemental Table S10. Serotype of obtained *H. influenzae* genome by hicap

isolate	predicted _serotype	genes_identified	locus_location	region_I_genes	region_II_genes	region_III_genes	IS1016_hits
consensus	type_f	hcsB,hcsA,fcs3^,fcs2^,fcs2^,fcs1*,fcs1*,bexD,bexC,bexB,bexA	1061133-1073560	4/4	1/3 (missing: fcs3,fcs2)	2/2	0

*truncation, ^blast hit only
