# Supplementary material for: Genetic Sequencing of a Bacterial Pneumonia Vaccine Produced in 1916
Source: Vaccines (Basel). 2025 May 2;13(5):491. doi: 10.3390/vaccines13050491 (PMC12115763; doi:10.3390/vaccines13050491)
Supplement: Supplementary file 1 [file vaccines-13-00491-s001.zip › SupplementalTableS11_MLST.pdf]

Supplemental Table S11. MLST typing of *E. faecium* genome at PubMLST at using 8 MLST genes (Bezdicek et al., 2023).

Locus	Allele	Length	Contig	Start position	End position
copA	6	518	*E. faecium*_consensus	2015693	2016210
dnaE	3	568	*E. faecium*_consensus	271293	271860
HP2027	3	581	*E. faecium*_consensus	1923883	1924463
mdlA	9	464	*E. faecium*_consensus	1941859	1942322
narB	3	403	*E. faecium*_consensus	1475377	1475779
rpoD	17	527	*E. faecium*_consensus	198423	198949
uvrA	2	694	*E. faecium*_consensus	2161873	2162566
