# Supplementary material for: Genetic Sequencing of a Bacterial Pneumonia Vaccine Produced in 1916
Source: Vaccines (Basel). 2025 May 2;13(5):491. doi: 10.3390/vaccines13050491 (PMC12115763; doi:10.3390/vaccines13050491)
Supplement: Supplementary file 1 [file vaccines-13-00491-s001.zip › SupplementalTableS1_RealtimePCR.pdf]

Supplemental Table S1. Real-time PCR of 16S rRNA gene of *Haemophilus influenzae*, *Streptococcus pneumoniae*, and *Streptococcus pyogenes*

Isolation From	Sample Name	16S gene	CT value
First ampule	PCR_PC[1]	*H. influenzae*	20.529
	PCR_PC	*S. pyogenes*	20.786
	PCR_PC	*S. pneumoniae*	19.542
	NTC[2]	*H. influenzae*	Undetermined
	NTC	*S. pyogenes*	Undetermined
	NTC	*S. pneumoniae*	Undetermined
	Microbial_PC[3]	*H. influenzae*	28.927
	Microbial_PC	*S. pyogenes*	32.081
	Microbial_PC	*S. pneumoniae*	29.750
	Supernatant_DNA[4]	*H. influenzae*	39.738
	Supernatant_DNA	*S. pyogenes*	Undetermined
	Supernatant_DNA	*S. pneumoniae*	Undetermined
	Precipitate_DNA [5]	*H. influenzae*	36.133
	Precipitate_DNA	*S. pyogenes*	39.915
	Precipitate_DNA	*S. pneumoniae*	Undetermined
Second ampule*	NTC	*H. influenzae*	Undetermined
	Microbial_PC	*H. influenzae*	29.982
	Supernatant_DNA	*H. influenzae*	38.545
	Precipitate_DNA	*H. influenzae*	35.312

[1]PCR_PC: Real-time PCR positive control. [2]NTC: Real-time PCR no-template control. [3]Microbial_PC: Real-time PCR positive control for microbial species. [4]Supernatant_DNA: DNA isolate from supernatant as template. [5]Precipitate_DNA: DNA isolate from precipitate as template. *From the second ampule, detection of only *H. influenzae* 16S gene was performed to save material.
