# Supplementary material for: Genetic Sequencing of a Bacterial Pneumonia Vaccine Produced in 1916
Source: Vaccines (Basel). 2025 May 2;13(5):491. doi: 10.3390/vaccines13050491 (PMC12115763; doi:10.3390/vaccines13050491)
Supplement: Supplementary file 1 [file vaccines-13-00491-s001.zip › SupplementalTableS2_Sequence.pdf]

Supplemental Table S2. Generated sequence from 2 vaccine vials on MiSeq and NextSeq 500.

Vaccine	Sequencer	DNA template	Read format	Read produced
First ampule	MiSeq	Pooled amplified DNA	150bp+150bp (paired end)	22,551,907
	NextSeq 500	Pooled amplified DNA	160bp (single end)	517,835,296
Second ampule	MiSeq	Nucleotide free dH_2O (Negative control)	100bp+8bp (single end + index)	10,199,050
	MiSeq	Pooled amplified DNA	100bp+8bp (single end + index)	15,368,293
	NextSeq 500	Pooled amplified DNA	100bp (single end)	416,537,815

Note: Single-end and shorter read lengths from the second ampule actually yielded better sequence read quality.
