# Supplementary figures and images for: Genetic Sequencing of a Bacterial Pneumonia Vaccine Produced in 1916
Source: Vaccines (Basel). 2025 May 2;13(5):491. doi: 10.3390/vaccines13050491 (PMC12115763; doi:10.3390/vaccines13050491)

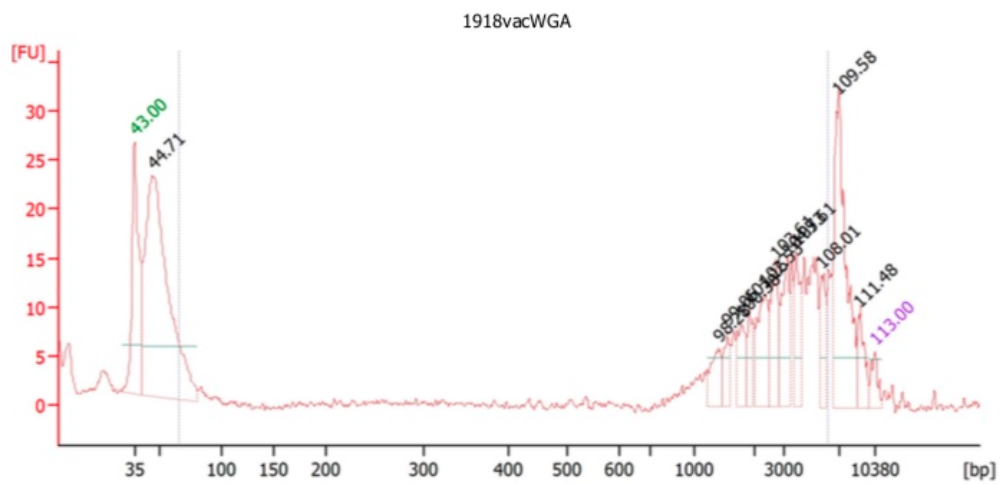

Supplement: Supplementary file 1 [file vaccines-13-00491-s001.zip › SupplementalFigureS1_WGA.pdf]
